# Supplementary material for: Obesity influences composition of salivary and fecal microbiota and impacts the interactions between bacterial taxa
Source: Physiol Rep. 2022 Apr 5;10(7):e15254. doi: 10.14814/phy2.15254 (PMC8980904; doi:10.14814/phy2.15254)
Supplement: Supplementary file 1 — Fig S1‐S4 [file PHY2-10-e15254-s001.pptx]

## Slide 1
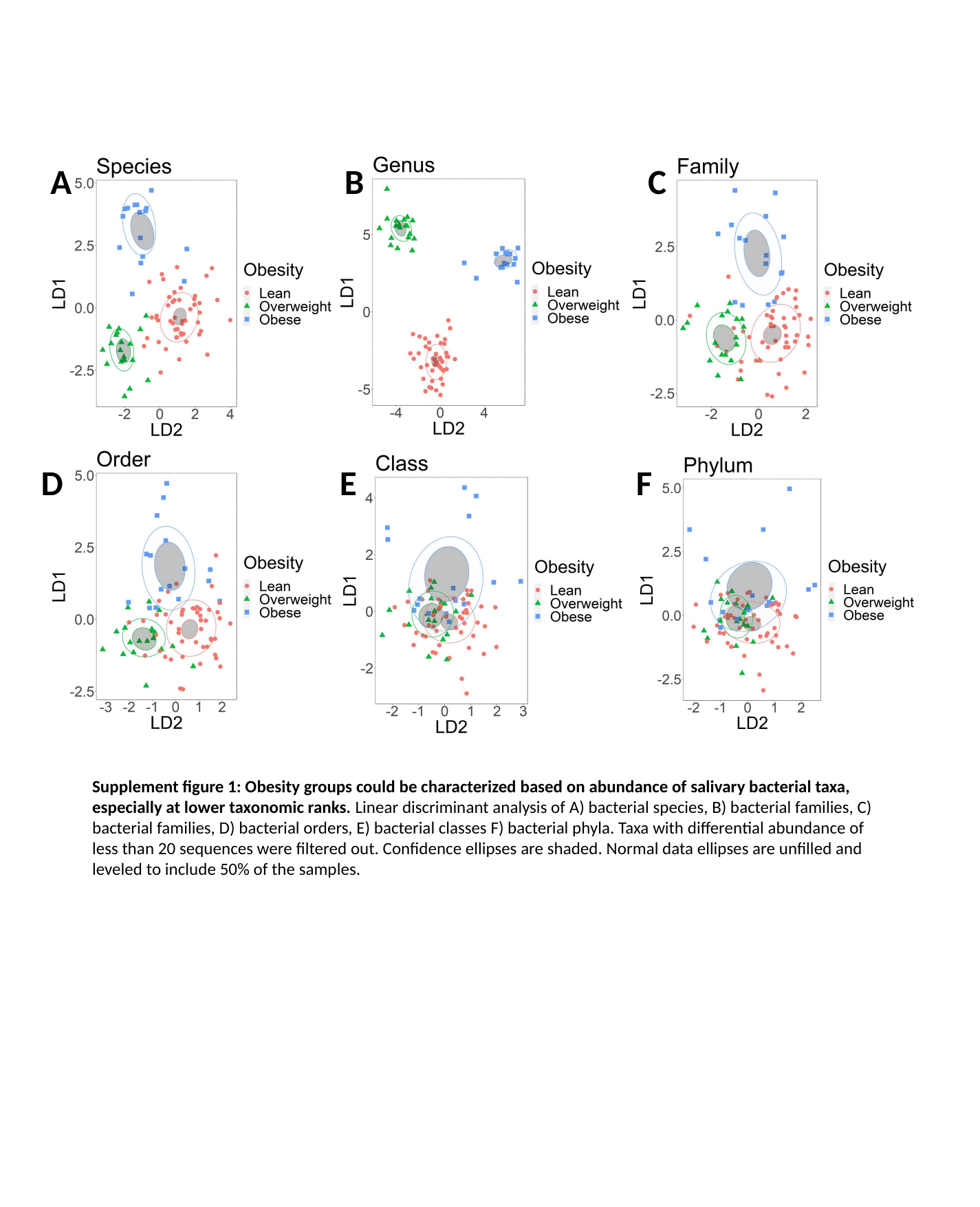

C
B
A
D
E
F
Supplement figure 1: Obesity groups could be characterized based on abundance of salivary bacterial taxa, especially at lower taxonomic ranks. Linear discriminant analysis of A) bacterial species, B) bacterial families, C) bacterial families, D) bacterial orders, E) bacterial classes F) bacterial phyla. Taxa with differential abundance of less than 20 sequences were filtered out. Confidence ellipses are shaded. Normal data ellipses are unfilled and leveled to include 50% of the samples.

## Slide 2
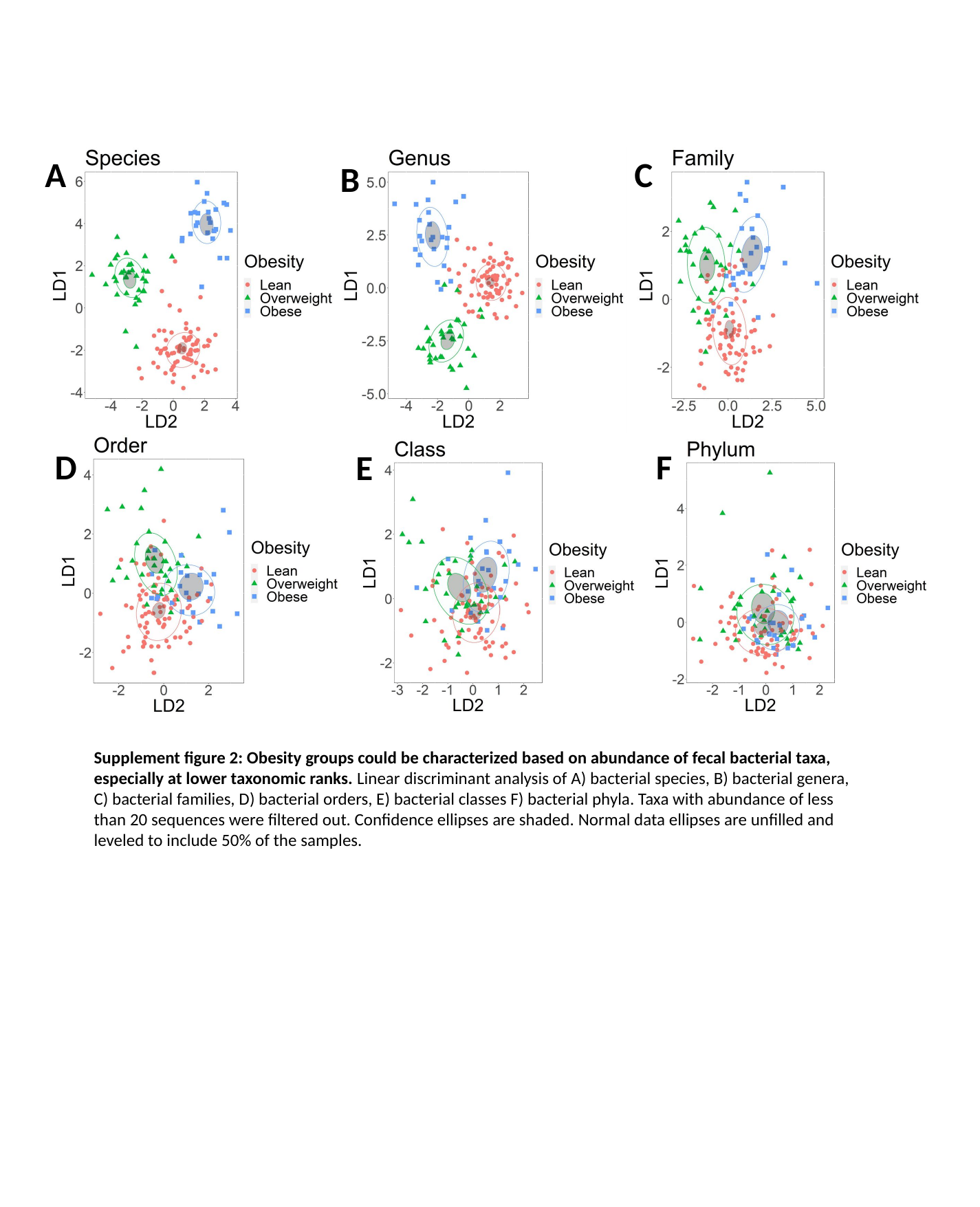

C
A
B
D
F
E
Supplement figure 2: Obesity groups could be characterized based on abundance of fecal bacterial taxa, especially at lower taxonomic ranks. Linear discriminant analysis of A) bacterial species, B) bacterial genera, C) bacterial families, D) bacterial orders, E) bacterial classes F) bacterial phyla. Taxa with abundance of less than 20 sequences were filtered out. Confidence ellipses are shaded. Normal data ellipses are unfilled and leveled to include 50% of the samples.

## Slide 3
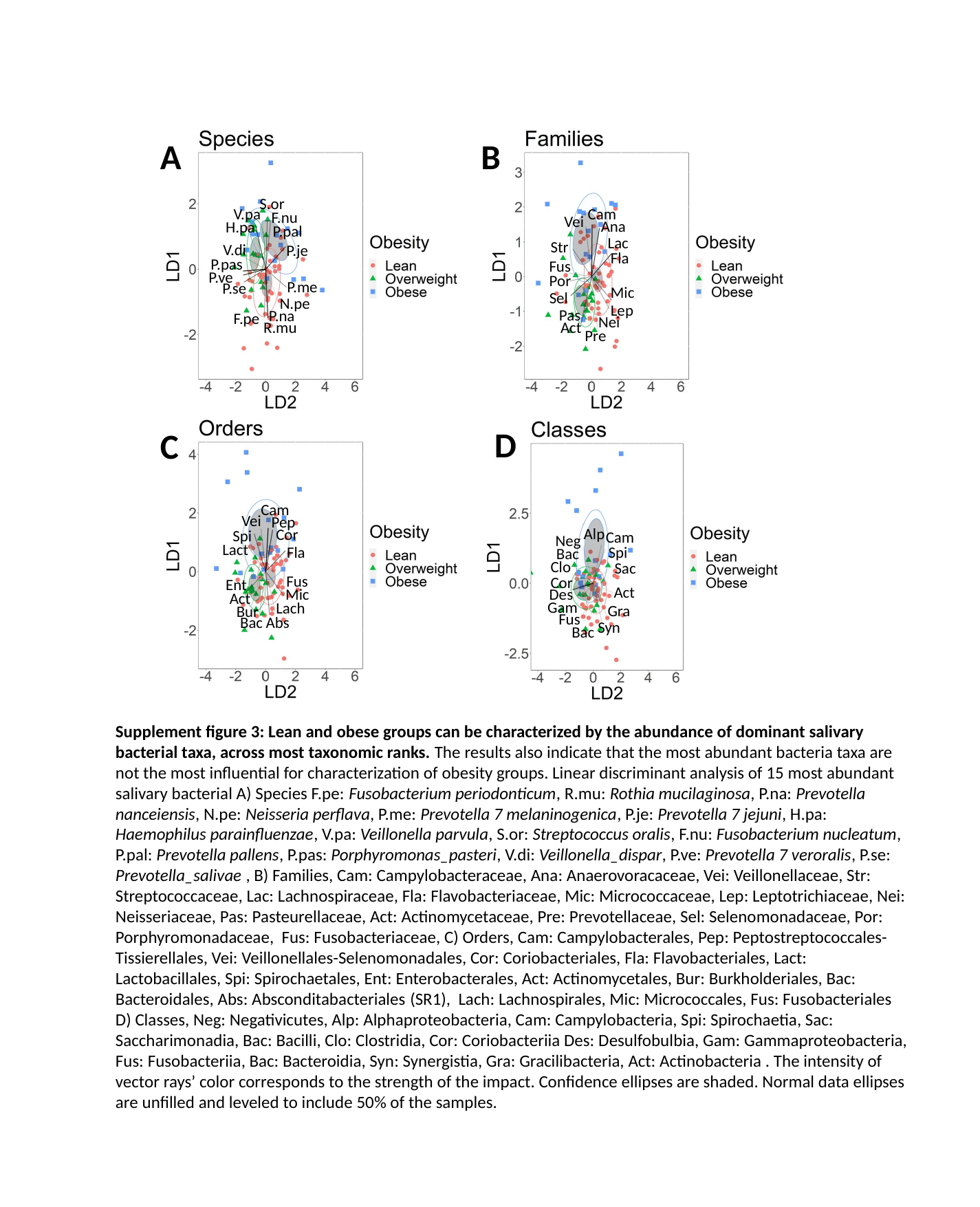

A
B
S.or
V.pa
Cam
F.nu
Vei
Ana
H.pa
P.pal
Lac
Str
V.di
P.je
Fla
P.pas
Fus
P.ve
Por
P.me
P.se
Mic
Sel
N.pe
Lep
Pas
P.na
F.pe
Nei
R.mu
Act
Pre
D
C
Cam
Vei
Pep
Alp
Cor
Spi
Cam
Neg
Lact
Fla
Spi
Bac
Clo
Sac
Fus
Cor
Ent
Act
Des
Mic
Act
Gam
Lach
Gra
Bur
Fus
Bac
Abs
Syn
Bac
Supplement figure 3: Lean and obese groups can be characterized by the abundance of dominant salivary bacterial taxa, across most taxonomic ranks. The results also indicate that the most abundant bacteria taxa are not the most influential for characterization of obesity groups. Linear discriminant analysis of 15 most abundant salivary bacterial A) Species F.pe: Fusobacterium periodonticum, R.mu: Rothia mucilaginosa, P.na: Prevotella nanceiensis, N.pe: Neisseria perflava, P.me: Prevotella 7 melaninogenica, P.je: Prevotella 7 jejuni, H.pa: Haemophilus parainfluenzae, V.pa: Veillonella parvula, S.or: Streptococcus oralis, F.nu: Fusobacterium nucleatum, P.pal: Prevotella pallens, P.pas: Porphyromonas_pasteri, V.di: Veillonella_dispar, P.ve: Prevotella 7 veroralis, P.se: Prevotella_salivae , B) Families, Cam: Campylobacteraceae, Ana: Anaerovoracaceae, Vei: Veillonellaceae, Str: Streptococcaceae, Lac: Lachnospiraceae, Fla: Flavobacteriaceae, Mic: Micrococcaceae, Lep: Leptotrichiaceae, Nei: Neisseriaceae, Pas: Pasteurellaceae, Act: Actinomycetaceae, Pre: Prevotellaceae, Sel: Selenomonadaceae, Por: Porphyromonadaceae, Fus: Fusobacteriaceae, C) Orders, Cam: Campylobacterales, Pep: Peptostreptococcales-Tissierellales, Vei: Veillonellales-Selenomonadales, Cor: Coriobacteriales, Fla: Flavobacteriales, Lact: Lactobacillales, Spi: Spirochaetales, Ent: Enterobacterales, Act: Actinomycetales, Bur: Burkholderiales, Bac: Bacteroidales, Abs: Absconditabacteriales (SR1), Lach: Lachnospirales, Mic: Micrococcales, Fus: Fusobacteriales D) Classes, Neg: Negativicutes, Alp: Alphaproteobacteria, Cam: Campylobacteria, Spi: Spirochaetia, Sac: Saccharimonadia, Bac: Bacilli, Clo: Clostridia, Cor: Coriobacteriia Des: Desulfobulbia, Gam: Gammaproteobacteria, Fus: Fusobacteriia, Bac: Bacteroidia, Syn: Synergistia, Gra: Gracilibacteria, Act: Actinobacteria . The intensity of vector rays’ color corresponds to the strength of the impact. Confidence ellipses are shaded. Normal data ellipses are unfilled and leveled to include 50% of the samples.

## Slide 4
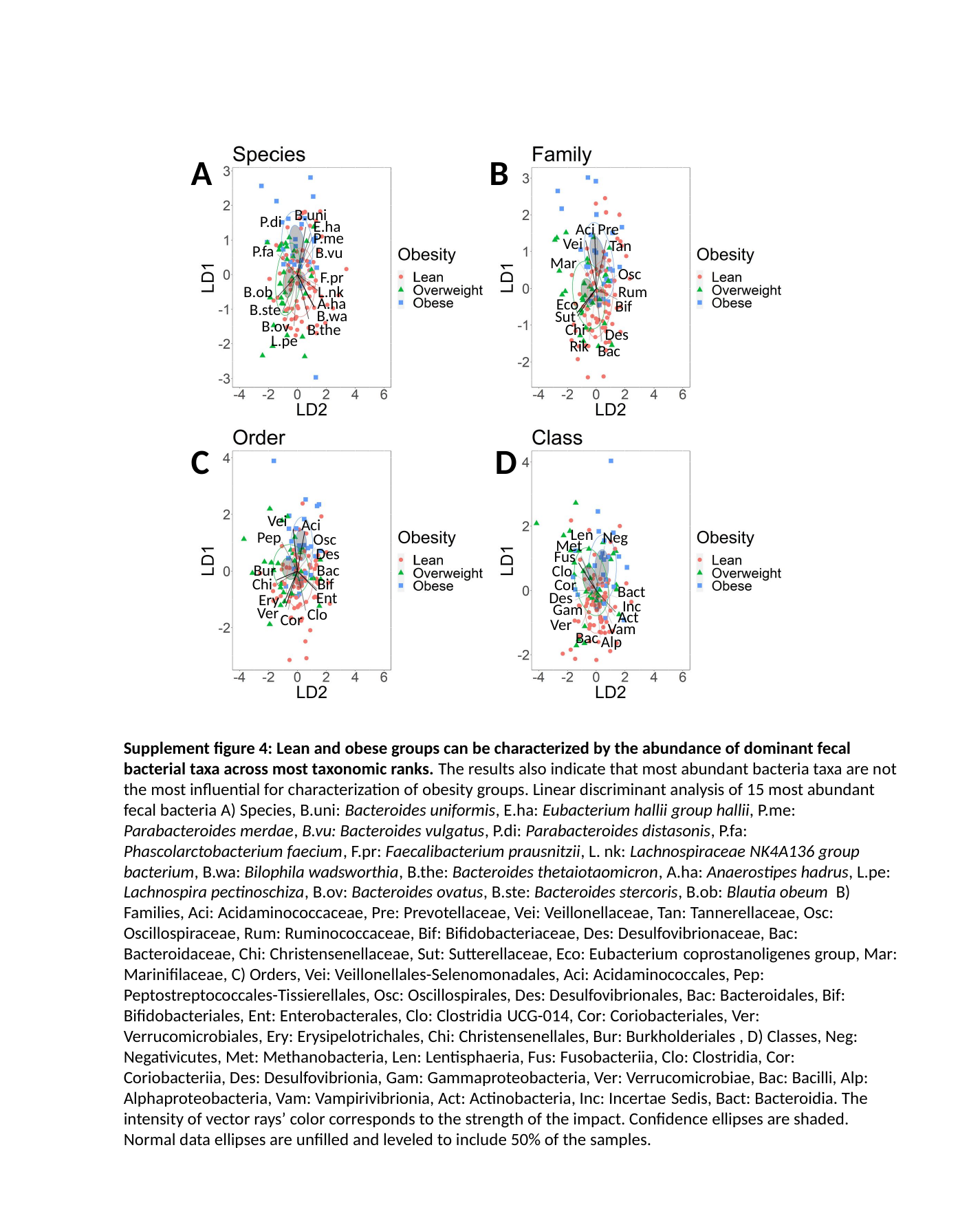

A
B
B.uni
P.di
E.ha
Aci
Pre
P.me
Vei
Tan
P.fa
B.vu
Mar
Osc
F.pr
B.ob
Rum
L.nk
A.ha
Eco
Bif
B.ste
B.wa
Sut
B.ov
B.the
Chi
Des
L.pe
Rik
Bac
D
C
Vei
Aci
Len
Pep
Neg
Osc
Met
Des
Fus
Bur
Bac
Clo
Bif
Chi
Cor
Bact
Des
Ent
Ery
Inc
Gam
Ver
Clo
Act
Cor
Ver
Vam
Bac
Alp
Supplement figure 4: Lean and obese groups can be characterized by the abundance of dominant fecal bacterial taxa across most taxonomic ranks. The results also indicate that most abundant bacteria taxa are not the most influential for characterization of obesity groups. Linear discriminant analysis of 15 most abundant fecal bacteria A) Species, B.uni: Bacteroides uniformis, E.ha: Eubacterium hallii group hallii, P.me: Parabacteroides merdae, B.vu: Bacteroides vulgatus, P.di: Parabacteroides distasonis, P.fa: Phascolarctobacterium faecium, F.pr: Faecalibacterium prausnitzii, L. nk: Lachnospiraceae NK4A136 group bacterium, B.wa: Bilophila wadsworthia, B.the: Bacteroides thetaiotaomicron, A.ha: Anaerostipes hadrus, L.pe: Lachnospira pectinoschiza, B.ov: Bacteroides ovatus, B.ste: Bacteroides stercoris, B.ob: Blautia obeum B) Families, Aci: Acidaminococcaceae, Pre: Prevotellaceae, Vei: Veillonellaceae, Tan: Tannerellaceae, Osc: Oscillospiraceae, Rum: Ruminococcaceae, Bif: Bifidobacteriaceae, Des: Desulfovibrionaceae, Bac: Bacteroidaceae, Chi: Christensenellaceae, Sut: Sutterellaceae, Eco: Eubacterium coprostanoligenes group, Mar: Marinifilaceae, C) Orders, Vei: Veillonellales-Selenomonadales, Aci: Acidaminococcales, Pep: Peptostreptococcales-Tissierellales, Osc: Oscillospirales, Des: Desulfovibrionales, Bac: Bacteroidales, Bif: Bifidobacteriales, Ent: Enterobacterales, Clo: Clostridia UCG-014, Cor: Coriobacteriales, Ver: Verrucomicrobiales, Ery: Erysipelotrichales, Chi: Christensenellales, Bur: Burkholderiales , D) Classes, Neg: Negativicutes, Met: Methanobacteria, Len: Lentisphaeria, Fus: Fusobacteriia, Clo: Clostridia, Cor: Coriobacteriia, Des: Desulfovibrionia, Gam: Gammaproteobacteria, Ver: Verrucomicrobiae, Bac: Bacilli, Alp: Alphaproteobacteria, Vam: Vampirivibrionia, Act: Actinobacteria, Inc: Incertae Sedis, Bact: Bacteroidia. The intensity of vector rays’ color corresponds to the strength of the impact. Confidence ellipses are shaded. Normal data ellipses are unfilled and leveled to include 50% of the samples.
